# Prokaryotic Community Structure of Long-Term Fertilization Field Andisols in Central Japan

**DOI:** 10.1128/MRA.01551-18

**Published:** 2019-01-17

**Authors:** Kazumori Mise, Hitoshi Moro, Takashi Kunito, Keishi Senoo, Shigeto Otsuka

**Affiliations:** a Department of Applied Biological Chemistry, Graduate School of Agricultural and Life Sciences, The University of Tokyo, Bunkyo-ku, Tokyo, Japan; b Department of Biological Sciences, Graduate School of Science, The University of Tokyo, Bunkyo-ku, Tokyo, Japan; c Department of Environmental Sciences, Faculty of Science, Shinshu University, Matsumoto, Nagano, Japan; d Collaborative Research Institute for Innovative Microbiology, The University of Tokyo, Bunkyo-ku, Tokyo, Japan; University of Maryland School of Medicine

## Abstract

Long-term fertilization experiments are a useful way to elucidate the impacts of fertilization on soil ecosystems. Here, we report the prokaryotic community structure in experimental field soil after 80 years of successive fertilization. Our 16S rRNA gene sequencing detected 20,996 amplicon sequence variants, including major phyla such as Proteobacteria, Acidobacteria, and Actinobacteria.

## Abstract (precis)

Long-term fertilization experiments are a useful way to elucidate the impacts of fertilization on soil ecosystems. Here, we report the prokaryotic community structure in experimental field soil after 80 years of successive fertilization.

## ANNOUNCEMENT

Fertilization management in arable soil is a fundamental focus in agricultural science. In Japanese volcanic ash soils (Andisols in USDA taxonomy), for example, heavy fertilization to enhance nutrient availability has long been conducted, resulting in an excessive accumulation of nutrients, and such fertilization practice likely affects soil microbial communities (1). However, the relationship between fertilization and soil microbial community dynamics is obscured in field experiments because soil microbial communities are affected by temporary soil management. As a preeminent solution to overcome this obstacle, we surveyed the prokaryotic community structure in a long-term fertilization experiment field, where fertilization has been consistently maintained for nearly eight decades.

**FIG 1:**
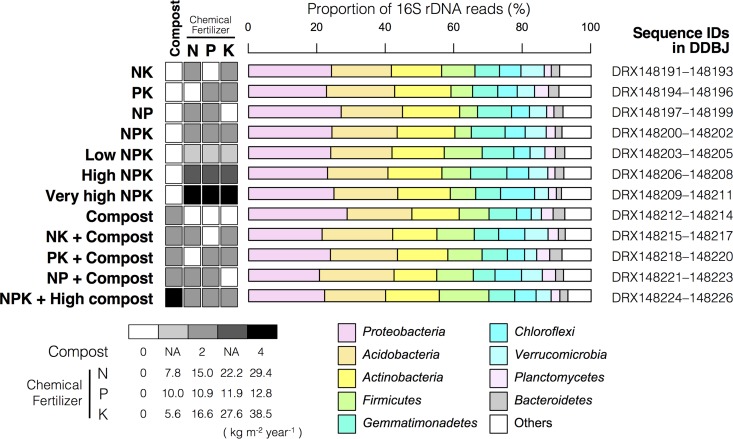
Fertilization treatment, taxonomic composition at the phylum level based on 16S rRNA gene sequencing, and corresponding DDBJ accession numbers of each sequence file. The presented taxonomic compositions are the average of three technical replicates. NA, not applicable.

The long-term fertilization experiment field is located in the Nagano Prefecture Vegetable and Ornamental Crops Experiment Station in Shiojiri, Nagano, Japan (36.10°N, 137.93°E). The soil is classified as an allophanic Andisol and light clay. Further details in soil texture, chemical, and biochemical properties have been previously reported (2). The fields were established in 1938, and the same fertilization management practices have been maintained. Ap soil horizons were sampled from 12 plots differing in fertilization management (Fig. 1). Soils were sieved through a 2-mm mesh and subjected to DNA extraction. Total DNA was extracted from 400 to 500 mg of soil using the FastDNA spin kit for soil (Qbiogene, Carlsbad, CA, USA). Before homogenizing soils and buffers, 50 µl of casein solution per 100 mg of soil (2% [wt/vol], dissolved in 300 mM sodium phosphate buffer [pH adjusted to 8.0]) was added to mitigate the DNA adsorption by soil allophane. The amplicon library was constructed as described by Caporaso et al. (3), with a modification that PCR was performed for 22 cycles. Briefly, the V4 region of the 16S rRNA gene was amplified with the 515F/806R primer pair, to which TruSeq adaptor and index sequences were attached. The size and concentration of the amplicons were checked by electrophoresis on agarose gels, and all amplicons were mixed together in equimolar amounts. The pooled amplicon was electrophoresed on an agarose gel and reextracted from the excised band using the Wizard SV gel and PCR clean-up system (Promega, Madison, WI, USA). The purified amplicon was quantified with a Qubit fluorometer (Thermo Fisher Scientific, Waltham, MA, USA) and subjected to 151-bp paired-end sequencing on a MiSeq version 2 reagent kit (Illumina, CA, USA), generating 334,310 to 1,641,608 reads per sample (23,054,242 reads in total). DNA extraction, amplicon library construction, and Illumina sequencing were conducted with three technical replicates for each soil sample. Sequences with ambiguous bases and low-quality sequences (expected errors of two bases or more) were removed. The filtered sequences were merged, and amplicon sequence variants (ASVs) and chimera-like ASVs were inferred using DADA2 version 1.10.0 (4) on R version 3.5.1 (5). Each nonchimeric ASV was taxonomically annotated by RDP Classifier version 2.3 (6) trained with Greengenes 13_8 (7) clustered at 97% sequence identity, followed by the removal of organelle-like (i.e., annotated either as “f__mitochondria” or “c__Chloroplast”) ASVs.

The yielded sequences that passed filtering ranged from 123,222 to 633,610 per sample, and 20,996 ASVs in total were obtained. The dominant phyla were mostly the same in all samples, namely, Proteobacteria (17.1 to 29.9%), Acidobacteria (16.3 to 23.4%), Actinobacteria (11.8 to 20.5%), Firmicutes (3.98 to 14.8%), Gemmatimonadetes (4.64 to 11.1%), Chloroflexi (4.14 to 10.3%), Verrucomicrobia (2.84 to 7.79%), Planctomycetes (1.73 to 5.94%), and Bacteroidetes (1.32 to 3.86%) (Fig. 1).

### Data availability.

The amplicon sequence data have been deposited in DDBJ under the accession number DRA007565.
